# Which beta-lactam infusion strategy is superior in ICU pneumonia? A Bayesian network meta-analysis of continuous, extended, and intermittent approaches

**DOI:** 10.1128/aac.01601-25

**Published:** 2026-03-16

**Authors:** Pei-Chun Lai, Yen-Ta Huang

**Affiliations:** 1 Education Centre, National Cheng Kung University Hospital, College of Medicine, National Cheng Kung University 34912, Tainan City, Taiwan; 2 Department of Paediatrics, National Cheng Kung University Hospital, College of Medicine, National Cheng Kung University 34912, Tainan City, Taiwan; 3 Department of Surgery, National Cheng Kung University Hospital, College of Medicine, National Cheng Kung University 34912, Tainan City, Taiwan; Providence Portland Medical Center, Portland, Oregon, USA

**Keywords:** beta-lactam, intermittent infusion, extended infusion, continuous infusion, Bayesian

## LETTER

We read with interest the systematic review and meta-analysis by Li et al. examining prolonged (continuous and extended) versus intermittent infusions of beta-lactam antibiotics in ICU patients with pneumonia (1). Based on frequentist analysis, the authors found no statistically significant improvements in two critical endpoints for severe infection: mortality (RR 0.79, 95% CI 0.59–1.07) and clinical cure rate (RR 1.09, 95% CI 0.99–1.21).

We identified several methodological considerations. First, many included RCTs contained sparse events or zero events in both arms, making risk difference (RD) within a Bayesian framework more appropriate than risk ratios (2, 3). RD also facilitates clearer communication of treatment effects to patients and families (4). Second, given inherent heterogeneity in patient severity and beta-lactam types, random-effects models should be preferred over the fixed-effects models used by the authors. Third, continuous and extended infusions might have sufficiently different pharmacokinetic profiles to warrant separate analysis. Finally, Bayesian approaches may be more suitable than frequentist p-values for critical care research, providing complementary insights for clinical decision-making (5).

**Fig 1:**
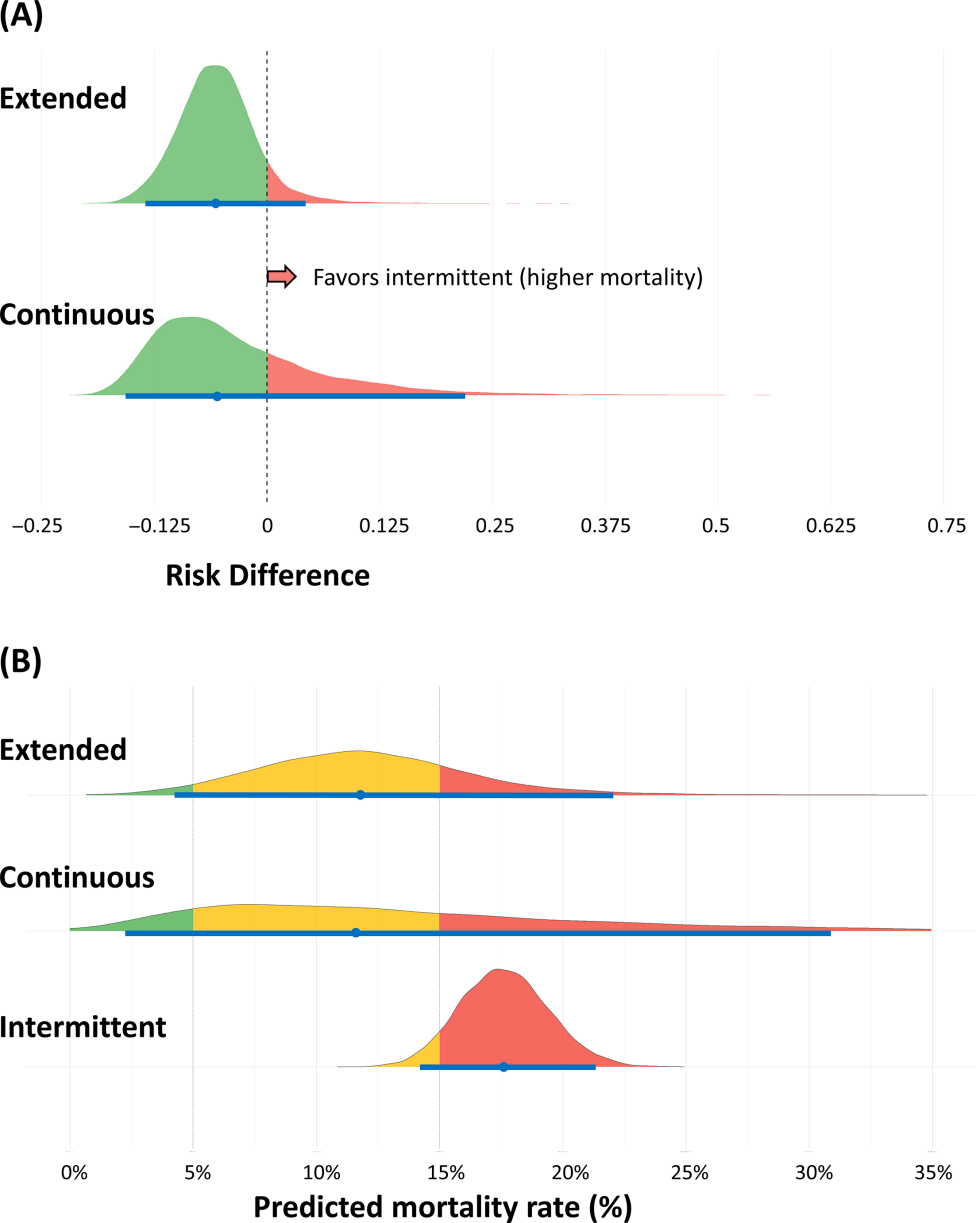
Bayesian network meta-analysis of mortality outcomes for beta-lactam infusion strategies in ICU patients with pneumonia. Half-eye plots show median (dot), 95% credible intervals (horizontal bars), and probability distributions. (**A**) Posterior distributions of risk differences for mortality comparing extended and continuous infusions versus intermittent infusion (reference). Green shading indicates probability of benefit (mortality reduction), while red shading indicates probability of harm. (**B**) Predicted absolute mortality rates for each infusion strategy. Colors indicate probability ranges: green (<5%), yellow (5%–15%), and red (>15%).

Therefore, we conducted a Bayesian network meta-analysis using multinma in R with random-effects models and non-informative priors. Models demonstrated convergence and good fit. The posterior distribution half-eye plot is shown in Fig. 1A. Compared with intermittent infusion, mortality RD was −0.0550 (95% credible interval [CrI] −0.155 to 0.227, 72.4% probability of benefit) for continuous infusion and −0.0573 (95% CrI −0.134 to 0.0514, 90.1% probability of benefit) for extended infusion. The surface under the cumulative ranking curve (SUCRA) analysis ranked extended infusion first (0.708), continuous second (0.605), and intermittent third (0.187). Predicted mortality (Fig. 1B) was lowest with extended infusion (11.8%, 95% CI 4.2%–23.4%), similar with continuous (11.9%, 95% CI 2.4%–40.4%), and highest with intermittent (17.6%, 95% CI 14.2%–21.3%). Notably, continuous infusion showed greater uncertainty due to limited RCTs with rare events. Intermittent infusion could not achieve mortality below 5% (0% probability) and had a 93.1% probability of mortality exceeding 15%.

**Fig 2:**
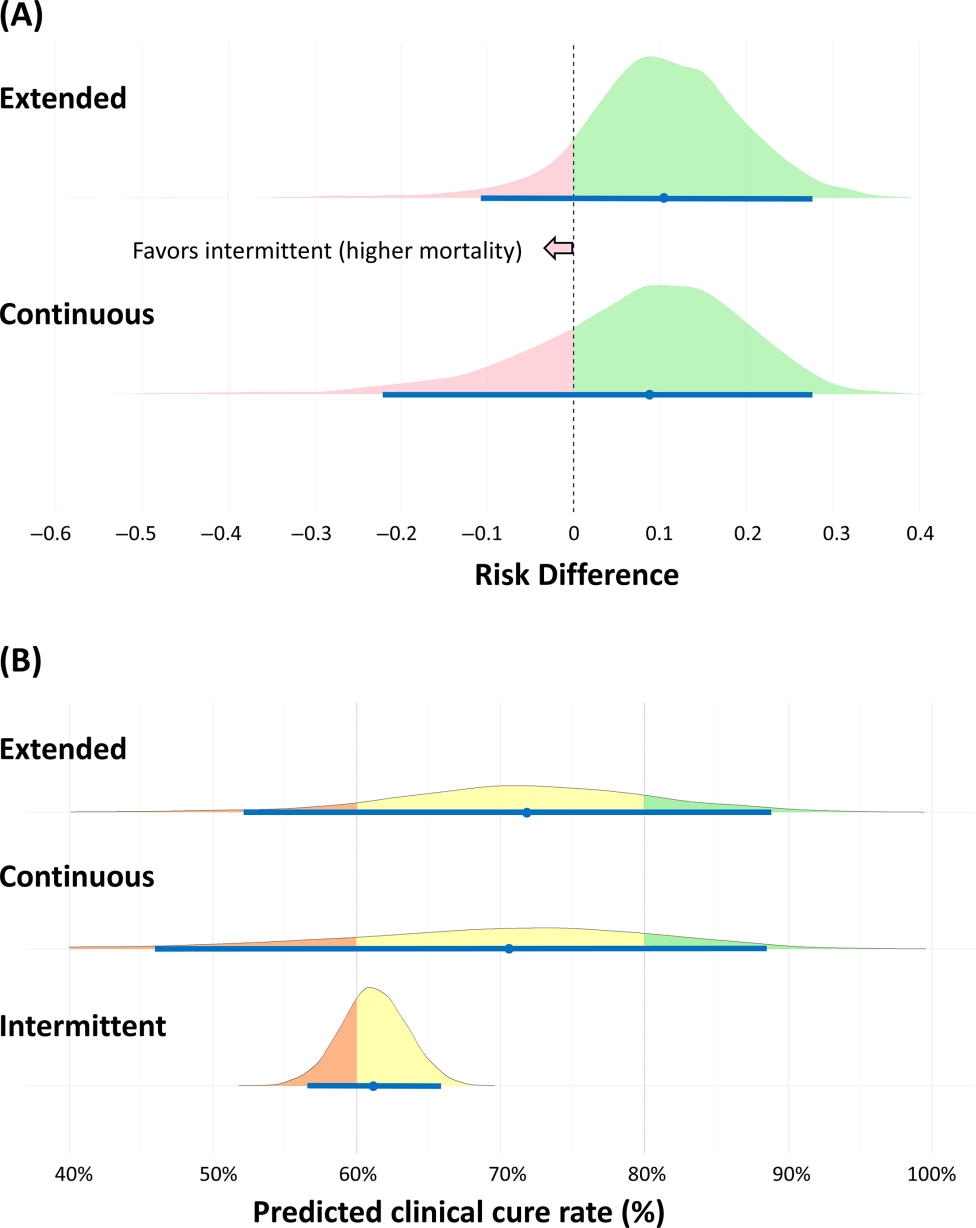
Bayesian network meta-analysis of clinical cure rate outcomes for beta-lactam infusion strategies in ICU patients with pneumonia. Half-eye plots display median (dot), 95% credible intervals (horizontal bars), and probability distributions. (**A**) Posterior distributions of risk differences for clinical cure rate comparing extended and continuous infusions versus intermittent infusion (reference). Light green shading represents probability of benefit (increased cure rate), while pink shading represents probability of reduced cure rate. (**B**) Predicted absolute clinical cure rates for each infusion strategy. Colors denote probability ranges: orange (<60%), yellow (60%–80%), and light green (>80%).

Clinical cure rates improved with both prolonged infusion strategies compared to intermittent infusion. Continuous infusion showed RD 0.0885 (95% CrI −0.221 to 0.270, 76.2% probability of benefit) and extended infusion showed RD 0.104 (95% CrI −0.106 to 0.274, 89.0% probability of benefit) (Fig. 2A). The consistent SUCRA ranking was extended (0.723), continuous (0.603), and intermittent (0.174). Predicted absolute clinical cure rates were 71.7% (95% CI 50.2%–88.9%) for extended, 70.3% (95% CI 38.5%–88.4%) for continuous, and 61.1% (95% CI 56.6%–65.7%) for intermittent infusion (Fig. 2B). Intermittent infusion could not achieve cure rates above 80% (0% probability) and had substantial risk (30.4%) of rates below 60%.

Through Bayesian network meta-analysis, both extended and continuous infusions demonstrated higher probability of improved outcomes compared to intermittent infusion for ICU pneumonia patients. Although effect estimates overlapped substantially, extended infusion showed slightly greater certainty and practical feasibility for implementation. We recommend incorporating Bayesian approaches alongside frequentist analyses in future meta-analyses, as they provide probabilistic interpretations valuable for clinical decision-making when traditional hypothesis testing may be less informative (6).
